# Fulton County Medical Society, Regular Meeting April 2, 1914

**Published:** 1914-04

**Authors:** R. R. Daly


### FULTON COUNTY MEDICAL SOCIETY.

Regular Meeting April 2, 1914.

By R. R. Daly, M. D.

Dr. R. R. Daly read a paper on "Tracheitis."

Dr. Newton Craig said that he had frequently seen the bifurcation of the trachea but that he had not used the same method as described in the paper. The trachea would show irritation along its whole length and it certainly needed local treatment. His own method was to use the intra-tracheal syringe and thus introduce a larger amount of the indicated solution than seemed possible with the applicator. This medication could be warmed and often times it was so bland that the patient would not know it had been injected.

He also spoke of a chronic form to tracheitis that simulated the conditions found in he nose afflicted with atrophic rhinitis, and said that this could be handled advantageously with the injections.

Dr. R. M. Nelson said that he had looked through the recent literature and text books and could find very little upon this subject. He said he had seen patients treated by Daly and approved of the method. There were times, however, when it could not be used and when sprays were contra-indicated. Then he had found a mixture of oil of pine needles m. 45, camphor gr. 45, methol grs. 90 and Tr. Benzoin 3 oz. of great assistance as used in a steam vaporizer. This was of value, too, in the corditis nodosa of singers. He suggested that as a routine of proceedure the larynx should be examined before other parts in the vicinity because otherwise, he preceeding gagging might cause factitious redness of the laryngeal tissues.

Dr. Thrash said that in tubercular throats, there was great sensitiveness and that the spasm resulting from the application might cause the cotton to be pulled off in the trachea.

Dr. Daly said in closing that as to the use of the syringe, he had found trouble in the sudden expulsion the patients sometimes made and had been showered with argyrol which was far from pleasant and profitable. Besides he could carry all he needed on an applicator and it seemed so much simpler and easier. Certainly it was quicker and efficatious. As to Dr. Thrash's criticism, he was careful always that the cotton could not come off and there was no danger whatever on that account. The method that Dr. Nelson spoke of was excellent and of great value in all throat cases when for any reason local applications were contra indicated.

Dr. W. F. Shallenberger showed some X-ray pictures of a patient whom he had carried through two important crises. The patient, a young woman, had attempted to abort herself at about the first menstrual absence. About a week later she had some pain in the right pelvis and down the thigh. Four weeks after the first trouble, she had total suppression of urine for twenty four hours. She looked sick and was in pain but there was no bleeding. Her condition was such that consultant advised emptying the uterus and an attempt was made to do this. It showed that there was an extra uterine prgnancy. An operation was performed and a mass of blood and clots was removed from the pelvis. There was great shock and the lady recovered only after having been kept in the Trendelenberg position for four hours. There was some cystitis following, but that disappeared.

A short time after recovery, there was an attack of renal colic and blood and pus showed in the right kidney. Four months later there were other attacks and a diagnosis of stone in the ureter was made. The X-ray disclosed the stone low down in the ureter. There were also what appeared to be smaller stones on the other side but a little farther from the middle line. The ureters were catheterizd and another picture taken. This showed the stones on the other side to be phleboliths in the broad ligament but in direct line antero-posteriorly with the ureter on that side. The stone on the right side was removed by crushing and through the use of the endoscope. The essentially interesting part of the case was the differentiation of the stone from the phleboliths.

Dr. Derr said that phleboliths in the pelvis are not uncommon, but they are always found further from the median line than ureteral obstructions.

Dr. J. L. Campbell reported a case of Extra-uterine Pregnancy with suppression of urine and death.

There had been no regular menstruation for 2½ months when the condition of the discharge called for curettage. This was done and afterward she failed to void. Catheterization gave 6 oz. 72 hours after the operation. Pelvic examination showed a mass about the size of an orange in the broad ligament. Operation disclosed ectopic pregnancy. The placenta was attached to the ovary, small intestine and convex surface of the sigmoid. The haemorrhage was hard to control. The kidneys secreted no urine and she died in 24 hours.

Dr. Battey asked if the chocolate colored discharge in extra uretrine cases comes from he tube or the uterus.

Dr. Shallenberger said that the discharge is characteristic and it probably come from both the uterus and the tubes.

Dr. J. L. Campbell showed two specimens of appendices he had removed, one in a man of 25 was after a moderate attack. The appendix was adherent to the caecum and in the caecum was a mass as large as a finger. Fearing malignancy he resected the whole mass. The patient recovered uneventfully. The other specimen was that of a tumor at the base of the appendix that demanded resection. It proved to be an abscess in the wall of the gut itself. Recovery.

Dr. S. T. Barnette said that he had had two interesting pelvic cases within the week. One was an appendix with pus in the cavity that did nicely and the other was a woman of 74 with rupture of the tube. She died.

Dr. J. S. Derr showed X-ray picture of unsuspected fracture of the surgical neck of the humerus. A dislocation was looked for but an impacted fracture was found. It was treated without breaking down the impaction and recovered good use.

Dr. Newton Craig reported a case of Ranula as large as his fist under the chin. It was opened from the outside because no duct could be found within. Foul secretions were evacuated and he hopes later to establish a connection between the gland and the mouth.

Dr. S. R. Roberts reported a case of Dry Mouth that came to him lately. The tongue was coatless and looked like the cross section of a fresh beet. Pellagra, uraemia, acute alcoholism and sprue were excluded. Also syphilis for in this disease, the tongue is cobble like. The kidneys gave a 50% output. It was diagnosed as a case of Xerostomia. There have been about 60 such cases reported. A one per cent. solution of nitrate of silver three times a week gives temporary relief. There is functional loss of power of the salivary glands.

He had another case to report. It was Maniacal Meningitis.

There were 3,400 pus cells to the c. m. m. in the cerebro spinal fluid. This excess fell to 800 after several punctures. She subsequently developed cyanosis and opsithotonos. This was due to the sudden increase in the spinal exudate following the punctures. It was relieved by the very slow withdrawal of 30 c.c. of the spinal fluid at the rate of a drop to 6 seconds. She recovered in 24 hours.

---

In abdominal surgery the field should be bloodless before closing the parietes. Don't depend upon it that "that little bleeding will stop," if a little extra pains with sutures or ligatures will make it stop. Sometimes "that little bleeding" doesn't stop, but causes an intraperitoneal hematoma or an alarming secondary hemorrhage.—American Journal of Surgery

